# (4-Bromo­phen­yl)(3,6-dimeth­oxy-2-naphth­yl)methanone

**DOI:** 10.1107/S160053681004016X

**Published:** 2010-10-13

**Authors:** Yuichi Kato, Atsushi Nagasawa, Kotaro Kataoka, Akiko Okamoto, Noriyuki Yonezawa

**Affiliations:** a Department of Organic and Polymer Materials Chemistry, Tokyo University of Agriculture & Technology, 2-24-16 Naka-machi, Koganei, Tokyo 184-8588, Japan

## Abstract

In the title compound, C_19_H_15_BrO_3_, the dihedral angle between the naphthalene ring system and the benzene ring is 62.51 (8)°. The bridging carbonyl C—C(=O)—C plane makes dihedral angles of 47.07 (6)° with the naphthalene ring system and 24.20 (10)° with the benzene ring. A weak inter­molecular C—H⋯O hydrogen bond exists between the H atom of one meth­oxy group and the O atom of the other meth­oxy group in an adjacent mol­ecule. The crystal packing is additionally stabilized by two types of weak inter­molecular inter­actions involving the Br atom, C—H⋯Br and Br⋯O [3.2802 (14) Å].

## Related literature

For electrophilic aromatic substitution of naphthalene deriv­atives affording *peri-*aroylated compounds regioselectively, see: Okamoto & Yonezawa (2009 ▶). For the structures of closely related compounds, see: Kato *et al.* (2010 ▶); Muto *et al.* (2010 ▶); Nakaema *et al.* (2008 ▶); Watanabe *et al.* (2010*a* ▶,*b* ▶).
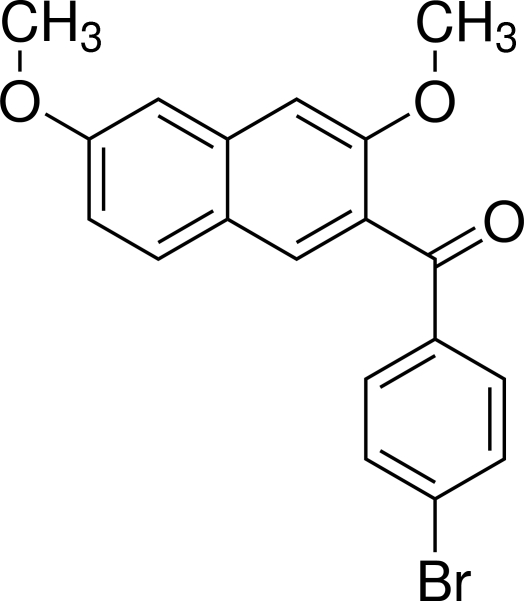


## Experimental

### 

#### Crystal data

C_19_H_15_BrO_3_
*M* *_r_* = 371.22
Monoclinic, 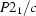

*a* = 7.88917 (14) Å
*b* = 21.0182 (4) Å
*c* = 10.06272 (18) Å
β = 105.971 (1)°
*V* = 1604.16 (5) Å^3^
*Z* = 4
Cu *K*α radiation
μ = 3.60 mm^−1^
*T* = 193 K
0.60 × 0.40 × 0.20 mm

#### Data collection

Rigaku R-AXIS RAPID diffractometer
Absorption correction: numerical (*NUMABS*; Higashi, 1999 ▶) *T*_min_ = 0.161, *T*_max_ = 0.533
29541 measured reflections
2934 independent reflections
2767 reflections with *I* > 2σ(*I*)
*R*_int_ = 0.044

#### Refinement

*R*[*F* ^2^ > 2σ(*F* ^2^)] = 0.032
*wR*(*F* ^2^) = 0.086
*S* = 1.12
2934 reflections
210 parameters
H-atom parameters constrained
Δρ_max_ = 0.43 e Å^−3^
Δρ_min_ = −1.03 e Å^−3^

### 

Data collection: *PROCESS-AUTO* (Rigaku, 1998 ▶); cell refinement: *PROCESS-AUTO*; data reduction: *CrystalStructure* (Rigaku/MSC, 2004 ▶); program(s) used to solve structure: *SIR2004* (Burla *et al.*, 2005 ▶); program(s) used to refine structure: *SHELXL97* (Sheldrick, 2008 ▶); molecular graphics: *ORTEPIII* (Burnett & Johnson, 1996 ▶); software used to prepare material for publication: *SHELXL97*.

## Supplementary Material

Crystal structure: contains datablocks I, global. DOI: 10.1107/S160053681004016X/vm2049sup1.cif

Structure factors: contains datablocks I. DOI: 10.1107/S160053681004016X/vm2049Isup2.hkl

Additional supplementary materials:  crystallographic information; 3D view; checkCIF report

## Figures and Tables

**Table 1:** Hydrogen-bond geometry (Å, °)

*D*—H⋯*A*	*D*—H	H⋯*A*	*D*⋯*A*	*D*—H⋯*A*
C19—H19*A*⋯O1^i^	0.96	2.53	3.477 (2)	170
C5—H5⋯Br^ii^	0.93	2.98	3.8441 (18)	155

Symmetry codes: (i) 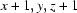; (ii) 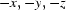.

**Fig. 1.**
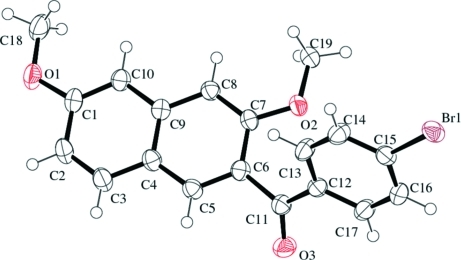
Molecular structure with displacement ellipsoids at 50% probability for non-H atoms.

**Fig. 2.**
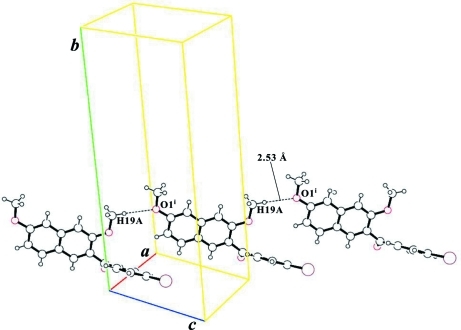
C—H···O interactions (dotted lines) [symmetry code: (i) x+1, y, z+1].

**Fig. 3.**
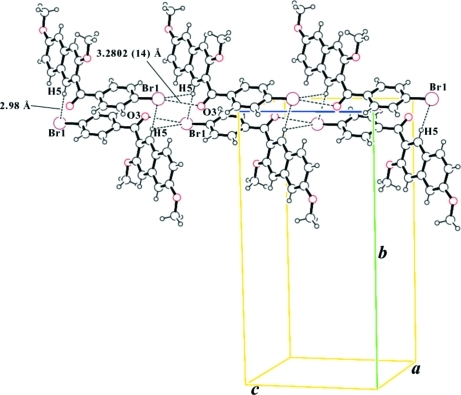
Two types of intermolecular weak interactions with bromine atom Br1 (dotted lines).

## supplementary crystallographic information

### Comment

In the course of our study on selective electrophilic aromatic aroylation of 2,7-dimethoxynaphthalene, *peri-*aroylnaphthalene compounds have proved to be formed regioselectively with the aid of suitable acidic mediator (Okamoto & Yonezawa, 2009). Recently, we reported the structures of 1,8-diaroyl-2,7-dimethoxynaphthalenes, i. e., 1,8-bis(4-methylbenzoyl)-2,7-dimethoxynaphthalene (Muto *et al.*, 2010), bis(4-bromophenyl)(2,7-dimethoxynaphthalene-1,8-diyl)dimethanone (Watanabe *et al.*, 2010*a*), and 1-aroyl-2,7-dimethoxynaphthalene, i. e., 1-benzoyl-2,7-dimethoxynaphthalene (Kato *et al.*, 2010). The aroyl groups at the 1,8-positions of the naphthalene rings in these compounds are twistedly bonded in a perpendicular manner but the benzene ring moieties of the aroyl groups tilt slightly toward the exo sides of the naphthalene rings. 1-Aroyl homologues also revealed essentially the same non-coplanar structure as observed for 1,8-diaroylated naphthalenes.

Furthermore, we also reported the crystal structure analysis of the corresponding *β*-isomers of 3-aroyl-2,7-dimethoxynaphthalenes such as 2-(4-chlorobenzoyl)-3,6-dimethoxynaphthalene (Nakaema *et al.*, 2008) and (3,6-dimethoxy-2-naphthyl)(4-fluorophenyl)methanone (Watanabe *et al.*, 2010*b*). In these 3-aroylated naphthalenes, which are generally regarded to be thermodynamically more stable than the corresponding 1-positioned isomeric molecules, the aroyl groups are shown connected to the naphthalene rings in a moderately twisted fashion. As part of our ongoing study on these homologous molecules, the synthesis and crystal structure of the title compound, a 3-monoaroylnaphthalene bearing a bromo group, is discussed in this article. The title compound was prepared by a direct condensation reaction of 2,7-dimethoxynaphthalene with 4-bromobenzoic acid.

The molecular structure of the title molecule is displayed in Fig. 1. The 4-bromophenyl group is bonded twistedly away from the attached naphthalene ring. The dihedral angle between the best planes of the bromophenyl ring (C12—C17) and the naphthalene ring (C1—C10) is 62.51 (8)°. The bridging carbonyl plane (O3—C6—C11—C12) makes a relatively large dihedral angle of 47.07 (9)° with the naphthalene ring (C1—C10) [C5—C6—C11—O3 torsion angle = 46.0 (2)°], whereas it makes a rather small angle of 24.20 (10)° with 4-bromophenyl ring (C12—C17) [O3—C11—C12—C17 torsion angle = 24.1 (3)°].

In the crystal structure, the molecular packing of the title compound is stabilized mainly by van der Waals interactions. Moreover, there is a C—H···O hydrogen bond between a hydrogen of the 2-methoxy group, which is situated adjacent to the bromophenyl group, and the ethereal oxygen atom of the 7-methoxy group in the neighboring molecule (Table 1, Fig. 2).

The crystal packing is additionally stabilized by two types of weak intermolecular interactions with the bromine atom: Br1···O3^i^ = 3.2802 (14) Å, and Br1···H5^ii^ = 2.98 Å [symmetry operations: (i) *x*, *y*, *z* + 1, (ii) -*x*, -*y*, -*z*] (Fig. 3) .

### Experimental

The title compound was prepared by treatment of a mixture of 2,7-dimethoxynaphthalene (1.88 g, 10 mmol) and 4-bromobenzoic acid (2.02 g, 10 mmol) with phosphorus pentoxide–methanesulfonic acid mixture (P_2_O_5_–MsOH [1/10 w/w]; 10 mL). After the reaction mixture was stirred at 353 K for 8 hours, the mixture was poured into ice-cooled water and extracted with CHCl_3_ (10 ml × 3). The combined extracts were washed with 2 *M* aqueous NaOH followed by washing with brine. The organic layer thus obtained was dried over anhydrous MgSO_4_. The solvent was removed under reduced pressure to give a cake (yield 3.07 g, 83%). The crude product was purified by flush silica gel chromatography (CHCl_3_). Colorless platelet single crystals suitable for X-ray diffraction were obtained by crystallization from ethanol and chloroform.

Spectroscopic Data:

^1^H NMR (300 MHz, CDCl_3_) δ 3.81 (3H, s), 3.93 (3H, s), 7.03 (1H, dd, 9.0 Hz), 7.09 (1H, d, J = 2.4 Hz), 7.12 (1H, s), 7.56 (2H, d, J = 8.4 Hz), 7.67-7.71 (3H, m), 7.78 (1H, s).

^13^C NMR (75 MHz, CDCl_3_) δ 55.32, 55.49, 105.01, 105.42, 117.16, 123.18, 127.32, 127.92, 130.05, 130.25, 131.30, 131.46, 136.96, 137.28, 155.64, 159.45, 194.94.

IR (KBr): 1626, 1585, 1501, 1134 cm^-1^.

HRMS (*m/z*): [*M* + H]^+^ calcd for C_19_H_16_BrO_3_, 371.0283; found, 371.0298.

### Refinement

All H atoms were found in a difference map and were subsequently refined as riding atoms, with C–H = 0.93 (aromatic) and 0.96 (methyl) Å, and with *U*_iso_(H) = 1.2*U*_eq_(C).

### Crystal data

**Table:**

C_19_H_15_BrO_3_	*F*(000) = 752
*M**_r_* = 371.22	*D*_x_ = 1.537 Mg m^−^^3^
Monoclinic, *P*2_1_/*c*	Melting point = 416.9–419.5 K
Hall symbol: -P 2ybc	Cu *K*α radiation, λ = 1.54187 Å
*a* = 7.88917 (14) Å	Cell parameters from 26572 reflections
*b* = 21.0182 (4) Å	θ = 4.2–68.3°
*c* = 10.06272 (18) Å	µ = 3.60 mm^−^^1^
β = 105.971 (1)°	*T* = 193 K
*V* = 1604.16 (5) Å^3^	Platelet, colorless
*Z* = 4	0.60 × 0.40 × 0.20 mm

### Data collection

**Table:**

Rigaku R-AXIS RAPID diffractometer	2934 independent reflections
Radiation source: rotating anode	2767 reflections with *I* > 2σ(*I*)
graphite	*R*_int_ = 0.044
Detector resolution: 10.00 pixels mm^-1^	θ_max_ = 68.3°, θ_min_ = 4.2°
ω scans	*h* = −9→9
Absorption correction: numerical (*NUMABS*; Higashi, 1999)	*k* = −25→25
*T*_min_ = 0.161, *T*_max_ = 0.533	*l* = −12→12
29541 measured reflections	

### Refinement

**Table:**

Refinement on *F*^2^	Primary atom site location: structure-invariant direct methods
Least-squares matrix: full	Secondary atom site location: difference Fourier map
*R*[*F*^2^ > 2σ(*F*^2^)] = 0.032	Hydrogen site location: inferred from neighbouring sites
*wR*(*F*^2^) = 0.086	H-atom parameters constrained
*S* = 1.12	*w* = 1/[σ^2^(*F*_o_^2^) + (0.0497*P*)^2^ + 0.6344*P*] where *P* = (*F*_o_^2^ + 2*F*_c_^2^)/3
2934 reflections	(Δ/σ)_max_ < 0.001
210 parameters	Δρ_max_ = 0.43 e Å^−^^3^
0 restraints	Δρ_min_ = −1.02 e Å^−^^3^

### Special details

**Table:**

Geometry. All e.s.d.'s (except the e.s.d. in the dihedral angle between two l.s. planes) are estimated using the full covariance matrix. The cell e.s.d.'s are taken into account individually in the estimation of e.s.d.'s in distances, angles and torsion angles; correlations between e.s.d.'s in cell parameters are only used when they are defined by crystal symmetry. An approximate (isotropic) treatment of cell e.s.d.'s is used for estimating e.s.d.'s involving l.s. planes.
Refinement. Refinement of *F*^2^ against ALL reflections. The weighted *R*-factor *wR* and goodness of fit *S* are based on *F*^2^, conventional *R*-factors *R* are based on *F*, with *F* set to zero for negative *F*^2^. The threshold expression of *F*^2^ > σ(*F*^2^) is used only for calculating *R*-factors(gt) *etc*. and is not relevant to the choice of reflections for refinement. *R*-factors based on *F*^2^ are statistically about twice as large as those based on *F*, and *R*- factors based on ALL data will be even larger.

### Fractional atomic coordinates and isotropic or equivalent isotropic displacement parameters (Å^2^)

**Table:**

	*x*	*y*	*z*	*U*_iso_*/*U*_eq_	
Br1	0.24719 (3)	0.056869 (10)	0.494126 (19)	0.03961 (11)	
O1	−0.46425 (17)	0.28009 (7)	−0.69867 (14)	0.0406 (3)	
O2	0.20106 (17)	0.19970 (6)	−0.05734 (13)	0.0342 (3)	
O3	0.24997 (19)	0.03129 (7)	−0.18371 (14)	0.0405 (3)	
C1	−0.3611 (2)	0.24042 (10)	−0.6033 (2)	0.0324 (4)	
C2	−0.3738 (3)	0.17558 (10)	−0.6441 (2)	0.0384 (4)	
H2	−0.4485	0.1636	−0.7292	0.046*	
C3	−0.2762 (3)	0.13090 (10)	−0.5582 (2)	0.0375 (4)	
H3	−0.2853	0.0885	−0.5854	0.045*	
C4	−0.1608 (2)	0.14784 (9)	−0.4282 (2)	0.0302 (4)	
C5	−0.0537 (2)	0.10319 (9)	−0.33881 (19)	0.0303 (4)	
H5	−0.0616	0.0605	−0.3643	0.036*	
C6	0.0620 (2)	0.12056 (9)	−0.21526 (18)	0.0284 (4)	
C7	0.0759 (2)	0.18618 (9)	−0.17702 (18)	0.0279 (4)	
C8	−0.0283 (2)	0.23056 (9)	−0.26096 (19)	0.0284 (4)	
H8	−0.0198	0.2730	−0.2340	0.034*	
C9	−0.1488 (2)	0.21296 (9)	−0.38808 (19)	0.0278 (4)	
C10	−0.2524 (2)	0.25890 (10)	−0.47798 (19)	0.0303 (4)	
H10	−0.2466	0.3015	−0.4520	0.036*	
C11	0.1740 (2)	0.06982 (9)	−0.1299 (2)	0.0308 (4)	
C12	0.1884 (2)	0.06594 (8)	0.02077 (19)	0.0291 (4)	
C13	0.0583 (2)	0.09052 (9)	0.0753 (2)	0.0349 (4)	
H13	−0.0401	0.1100	0.0169	0.042*	
C14	0.0731 (3)	0.08652 (9)	0.2154 (2)	0.0363 (4)	
H14	−0.0155	0.1024	0.2509	0.044*	
C15	0.2217 (3)	0.05857 (8)	0.3014 (2)	0.0312 (4)	
C16	0.3515 (3)	0.03239 (10)	0.2499 (2)	0.0370 (4)	
H16	0.4493	0.0127	0.3087	0.044*	
C17	0.3339 (3)	0.03590 (10)	0.1099 (2)	0.0358 (4)	
H17	0.4200	0.0180	0.0743	0.043*	
C18	−0.4465 (3)	0.34656 (10)	−0.6723 (2)	0.0454 (5)	
H18A	−0.5287	0.3692	−0.7451	0.055*	
H18B	−0.3286	0.3596	−0.6683	0.055*	
H18C	−0.4704	0.3558	−0.5857	0.055*	
C19	0.2347 (2)	0.26549 (10)	−0.0224 (2)	0.0348 (4)	
H19A	0.3294	0.2689	0.0609	0.042*	
H19B	0.1305	0.2847	−0.0086	0.042*	
H19C	0.2667	0.2869	−0.0962	0.042*	

### Atomic displacement parameters (Å^2^)

**Table:**

	*U*^11^	*U*^22^	*U*^33^	*U*^12^	*U*^13^	*U*^23^
Br1	0.05907 (18)	0.03269 (17)	0.02418 (16)	0.00179 (8)	0.00659 (11)	−0.00036 (7)
O1	0.0367 (7)	0.0454 (8)	0.0321 (7)	0.0022 (6)	−0.0032 (6)	0.0083 (6)
O2	0.0389 (7)	0.0309 (7)	0.0260 (7)	0.0006 (5)	−0.0024 (5)	−0.0014 (5)
O3	0.0488 (8)	0.0407 (8)	0.0321 (7)	0.0134 (6)	0.0113 (6)	−0.0007 (6)
C1	0.0269 (8)	0.0411 (11)	0.0274 (9)	0.0000 (7)	0.0044 (7)	0.0071 (8)
C2	0.0370 (9)	0.0451 (12)	0.0269 (10)	−0.0061 (8)	−0.0017 (8)	0.0000 (8)
C3	0.0399 (10)	0.0365 (11)	0.0309 (11)	−0.0069 (8)	0.0011 (8)	−0.0009 (9)
C4	0.0301 (8)	0.0321 (10)	0.0278 (9)	−0.0050 (7)	0.0071 (7)	0.0003 (7)
C5	0.0353 (9)	0.0280 (9)	0.0272 (9)	−0.0032 (7)	0.0077 (7)	−0.0001 (7)
C6	0.0310 (8)	0.0298 (9)	0.0248 (9)	0.0001 (7)	0.0083 (7)	0.0030 (7)
C7	0.0287 (8)	0.0325 (9)	0.0216 (8)	−0.0019 (7)	0.0055 (6)	−0.0005 (7)
C8	0.0308 (8)	0.0271 (9)	0.0263 (9)	−0.0003 (7)	0.0066 (7)	−0.0001 (7)
C9	0.0261 (8)	0.0320 (10)	0.0260 (9)	−0.0010 (7)	0.0083 (7)	0.0028 (7)
C10	0.0298 (9)	0.0326 (10)	0.0282 (9)	−0.0004 (7)	0.0074 (7)	0.0036 (7)
C11	0.0324 (9)	0.0292 (9)	0.0292 (10)	0.0004 (7)	0.0060 (7)	0.0010 (8)
C12	0.0331 (9)	0.0248 (9)	0.0282 (10)	0.0013 (7)	0.0063 (7)	0.0023 (7)
C13	0.0339 (9)	0.0383 (10)	0.0311 (10)	0.0102 (8)	0.0067 (8)	0.0084 (8)
C14	0.0404 (10)	0.0361 (10)	0.0337 (10)	0.0087 (8)	0.0123 (8)	0.0054 (8)
C15	0.0427 (10)	0.0243 (9)	0.0246 (9)	−0.0018 (7)	0.0059 (8)	0.0015 (7)
C16	0.0371 (9)	0.0375 (11)	0.0313 (10)	0.0092 (8)	0.0006 (8)	0.0032 (8)
C17	0.0358 (9)	0.0372 (11)	0.0335 (10)	0.0107 (8)	0.0081 (8)	0.0030 (9)
C18	0.0429 (11)	0.0452 (12)	0.0415 (12)	0.0071 (9)	0.0004 (9)	0.0127 (10)
C19	0.0362 (9)	0.0335 (11)	0.0304 (9)	−0.0010 (7)	0.0019 (8)	−0.0052 (8)

### Geometric parameters (Å, °)

**Table:**

Br1—C15	1.8942 (19)	C8—H8	0.9300
O1—C1	1.359 (2)	C9—C10	1.418 (3)
O1—C18	1.422 (3)	C10—H10	0.9300
O2—C7	1.361 (2)	C11—C12	1.491 (3)
O2—C19	1.433 (2)	C12—C13	1.390 (3)
O3—C11	1.219 (2)	C12—C17	1.398 (3)
C1—C10	1.372 (3)	C13—C14	1.384 (3)
C1—C2	1.419 (3)	C13—H13	0.9300
C2—C3	1.362 (3)	C14—C15	1.383 (3)
C2—H2	0.9300	C14—H14	0.9300
C3—C4	1.419 (3)	C15—C16	1.383 (3)
C3—H3	0.9300	C16—C17	1.379 (3)
C4—C5	1.409 (3)	C16—H16	0.9300
C4—C9	1.423 (3)	C17—H17	0.9300
C5—C6	1.374 (3)	C18—H18A	0.9600
C5—H5	0.9300	C18—H18B	0.9600
C6—C7	1.428 (3)	C18—H18C	0.9600
C6—C11	1.496 (3)	C19—H19A	0.9600
C7—C8	1.370 (3)	C19—H19B	0.9600
C8—C9	1.417 (2)	C19—H19C	0.9600
			
C1—O1—C18	117.51 (15)	O3—C11—C6	120.23 (18)
C7—O2—C19	117.30 (14)	C12—C11—C6	119.37 (16)
O1—C1—C10	125.22 (19)	C13—C12—C17	118.65 (18)
O1—C1—C2	113.82 (17)	C13—C12—C11	121.55 (16)
C10—C1—C2	120.96 (17)	C17—C12—C11	119.78 (17)
C3—C2—C1	119.73 (18)	C14—C13—C12	121.02 (17)
C3—C2—H2	120.1	C14—C13—H13	119.5
C1—C2—H2	120.1	C12—C13—H13	119.5
C2—C3—C4	121.30 (19)	C15—C14—C13	118.84 (18)
C2—C3—H3	119.4	C15—C14—H14	120.6
C4—C3—H3	119.4	C13—C14—H14	120.6
C5—C4—C3	122.78 (18)	C14—C15—C16	121.49 (18)
C5—C4—C9	118.58 (16)	C14—C15—Br1	118.80 (15)
C3—C4—C9	118.61 (17)	C16—C15—Br1	119.71 (14)
C6—C5—C4	122.20 (17)	C17—C16—C15	119.00 (17)
C6—C5—H5	118.9	C17—C16—H16	120.5
C4—C5—H5	118.9	C15—C16—H16	120.5
C5—C6—C7	118.89 (16)	C16—C17—C12	120.94 (18)
C5—C6—C11	118.06 (17)	C16—C17—H17	119.5
C7—C6—C11	122.98 (16)	C12—C17—H17	119.5
O2—C7—C8	124.72 (16)	O1—C18—H18A	109.5
O2—C7—C6	115.05 (15)	O1—C18—H18B	109.5
C8—C7—C6	120.19 (16)	H18A—C18—H18B	109.5
C7—C8—C9	121.22 (17)	O1—C18—H18C	109.5
C7—C8—H8	119.4	H18A—C18—H18C	109.5
C9—C8—H8	119.4	H18B—C18—H18C	109.5
C8—C9—C10	121.58 (18)	O2—C19—H19A	109.5
C8—C9—C4	118.88 (16)	O2—C19—H19B	109.5
C10—C9—C4	119.50 (17)	H19A—C19—H19B	109.5
C1—C10—C9	119.90 (19)	O2—C19—H19C	109.5
C1—C10—H10	120.1	H19A—C19—H19C	109.5
C9—C10—H10	120.1	H19B—C19—H19C	109.5
O3—C11—C12	120.38 (17)		
			
C18—O1—C1—C10	6.0 (3)	C3—C4—C9—C10	0.4 (3)
C18—O1—C1—C2	−173.88 (17)	O1—C1—C10—C9	−178.98 (17)
O1—C1—C2—C3	179.56 (18)	C2—C1—C10—C9	0.9 (3)
C10—C1—C2—C3	−0.3 (3)	C8—C9—C10—C1	176.76 (17)
C1—C2—C3—C4	−0.2 (3)	C4—C9—C10—C1	−0.9 (2)
C2—C3—C4—C5	−177.87 (19)	C5—C6—C11—O3	46.0 (3)
C2—C3—C4—C9	0.2 (3)	C7—C6—C11—O3	−131.1 (2)
C3—C4—C5—C6	177.68 (18)	C5—C6—C11—C12	−132.63 (18)
C9—C4—C5—C6	−0.3 (3)	C7—C6—C11—C12	50.3 (2)
C4—C5—C6—C7	−0.9 (3)	O3—C11—C12—C13	−154.64 (19)
C4—C5—C6—C11	−178.08 (17)	C6—C11—C12—C13	24.0 (3)
C19—O2—C7—C8	−4.8 (2)	O3—C11—C12—C17	24.1 (3)
C19—O2—C7—C6	172.93 (15)	C6—C11—C12—C17	−157.30 (19)
C5—C6—C7—O2	−176.14 (16)	C17—C12—C13—C14	1.1 (3)
C11—C6—C7—O2	0.9 (2)	C11—C12—C13—C14	179.87 (18)
C5—C6—C7—C8	1.7 (3)	C12—C13—C14—C15	1.2 (3)
C11—C6—C7—C8	178.79 (16)	C13—C14—C15—C16	−2.7 (3)
O2—C7—C8—C9	176.31 (16)	C13—C14—C15—Br1	176.93 (15)
C6—C7—C8—C9	−1.4 (3)	C14—C15—C16—C17	1.8 (3)
C7—C8—C9—C10	−177.62 (16)	Br1—C15—C16—C17	−177.86 (15)
C7—C8—C9—C4	0.1 (3)	C15—C16—C17—C12	0.7 (3)
C5—C4—C9—C8	0.8 (3)	C13—C12—C17—C16	−2.1 (3)
C3—C4—C9—C8	−177.36 (17)	C11—C12—C17—C16	179.15 (19)
C5—C4—C9—C10	178.52 (16)		

### Hydrogen-bond geometry (Å, °)

**Table:**

*D*—H···*A*	*D*—H	H···*A*	*D*···*A*	*D*—H···*A*
C19—H19A···O1^i^	0.96	2.53	3.477 (2)	170
C5—H5···Br^ii^	0.93	2.98	3.8441 (18)	155

Symmetry codes: (i) *x*+1, *y*, *z*+1; (ii) −*x*, −*y*, −*z*.
